# Influence of design and material characteristics on 3D printed flow-cells for heat transfer-based analytical devices

**DOI:** 10.1007/s00604-022-05163-2

**Published:** 2022-01-24

**Authors:** Leonardo F. Figueiredo, Felipe S. Vieira, Oliver D. Jamieson, Jack Reeder, Thomas Mc Lean, Jennifer Olsen, Robert D. Crapnell, Matthew J. Whittingham, Craig E. Banks, Richard Law, Jonas Gruber, Marloes Peeters

**Affiliations:** 1 Faculdade de Ciências Farmacêuticas, Universidade de São Paulo, Av. Prof. Lineu Prestes, 580, São Paulo, SP CEP 05508-000 Brazil; 2 Departamento de Engenharia Química, Escola Politécnica, Universidade de São Paulo, Avenida Prof. Luciano Gualberto, trav. 3, 380, São Paulo, SP CEP 05508-900 Brazil; 3 School of Engineering, Merz Court, Newcastle University, Claremont Road, Newcastle Upon Tyne, NE1 7RU UK; 4 Faculty of Science and Engineering, Manchester Metropolitan University, John Dalton Building, Chester Street, Manchester, M1 5GD UK; 5 Departamento de Química Fundamental, Instituto de Química, Universidade de São Paulo, Av. Prof. Lineu Prestes, 748, São Paulo, SP CEP 05508-000 Brazil

**Keywords:** Additive manufacturing, 3D printing, Biomimetic sensors, Molecularly imprinted polymers (MIPs), Heat transfer method (HTM), Antibiotics, Antimicrobial resistance

## Abstract

Redesigning 3D-printed flow cells is reported used for heat transfer based detection of biomolecules from a flow-through system to an addition-type measurement cell. The aim of this study is to assess the performance of this new measurement design and critically analyse the influence of material properties and 3D printing approach on thermal analysis. Particular attention is paid to reduce the time to stabilisation, the sample volume in order to make the technique suitable for clinical applications, and improving the sensitivity of the platform by decreasing the noise and interference of air bubbles. The three different approaches that were studied included a filament polylactic acid cell using only fused filament fabrication (FFF), a resin cell printed using stereolitography (SLA), and finally a design made of copper, which was manufactured by combining metal injection moulding (MIM) with fused filament fabrication (FFF). Computational fluid dynamic (CFD) modelling was undertaken using ANSYS Fluent V18.1 to provide insight into the flow of heat within the measurement cell, facilitating optimisation of the system and theoretical response speed.

It was shown that the measurement cells using SLA had the lowest noise (~ 0.6%) and shortest measurement time (15 min), whereas measurement cells produced using other approaches had lower specificity or suffered from voiding issues. Finally, we assessed the potential of these new designs for detection of biomolecules and amoxicillin, a commonly used beta lactam antibiotic, to demonstrate the proof of concept. It can be concluded that the resin addition-type measurement cells produced with SLA are an interesting affordable alternative, which were able to detect amoxicillin with high sensitivity and have great promise for clinical applications due to the disposable nature of the measurement cells in addition to small sample volumes.

**Graphical abstract:**

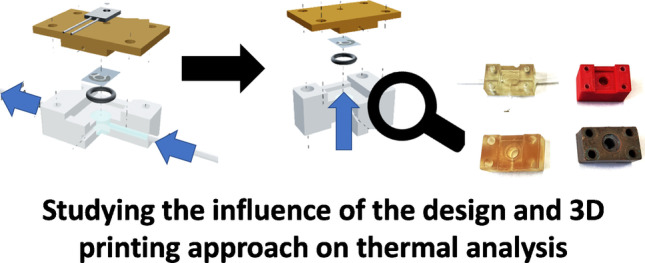

**Supplementary Information:**

The online version contains supplementary material available at 10.1007/s00604-022-05163-2.

## Introduction

The heat transfer method has emerged as an appealing sensing strategy due to its low-cost, fast and label-free nature [1]. When combined with selective receptors, it is possible to develop highly sensitive biosensors [2]. In the biosensing community, there is a strong drive for sensor platforms with improved sensitivity, selectivity and reproducibility, in order to meet stringent requirements needed to apply this technology for medical diagnostics. Therefore, it has fuelled the drive for optimisation of all aspects of the devices rather than focusing on solely the recognition elements. In this manuscript, we will optimise the detection of amoxicillin using molecularly imprinted polymers (MIPs) as synthetic receptors combined with the heat-transfer method (HTM) as a read-out technique by evaluating different measurement cells made of different materials and produced by various additive manufacturing approaches.

HTM is based on analysing the heat transfer of a solid–liquid interface, which can be a functionalised electrode or thermocouple that is inserted into a measurement cell [3]. This method has been used for a range of applications starting from DNA mutation analysis [4] and was expanded to small molecule detection [5, 6], protein sensing [7] and bacteria identification [8] by using MIPs as biomimetic recognition elements. MIPs are porous materials that are able to rebind their respective target molecules with high specificity and selectivity, which offer the advantages of low-cost, robustness and versatility over natural recognition elements [9, 10]. It has been reported that the original design of the flow cell, which featured a large copper heat sink, led to considerable heat loss to the environment that imposes significant noise on the thermal signal [11]. Stilman et al. [12] designed a flow cell that optimised detection by focusing heat flow through the functionalised interface and measuring the temperature closer to the sensor surface. The dimensions of the liquid basin also have a significant impact; computational fluid dynamic modelling demonstrated that an elongated reservoir leads to reducing fluctuation in the signal and fewer “dead” zones in the cell. Subsequent experimental results with molecularly imprinted nanoparticles (nanoMIPs) confirmed these findings, and the overall detection of emerging cardiac biomarkers was improved [13]. The Wagner group [14] introduced the “hot-wire” concept, where functionalised aluminium wires served simultaneously as heating element and temperature sensors. The wire is heated periodically with an alternating current and thereby minimises parasitic heat loss that was inherent to previous thermal-resistance biosensors. An alternative option to minimise sample volumes of thermal analysis includes the use of transient plane source technology to measure the thermal conductivity of materials [15], which has been integrated into wearable sensors to measure the moisture content [16]. Furthermore, it is possible to replace thermocouples with thermistors, which exhibit higher sensitivity in specific temperature ranges and can be miniaturised [17].

Currently employed flow cells to monitor heat transfer resistance at the solid–liquid interface mostly have a “flow-through” system where a programmable syringe pump is utilised that replaces all liquid within the flow cell. The drawbacks of these methods include measurement drift and introducing disturbance, such as air bubbles, which impacts on the noise, and requiring a much higher sample volume compared to the inner volume of the flow cell. The designs in this study compare the original (“flow-through”) design to a novel (“addition-type”) measurement set up produced with different methods of additive manufacturing.

The flow cells produced were made via stereolitography (SLA), which requires the use of a resin that is photopolymerised [18] and offers the best resolution for achieving a design that contains inlets for tubing. The reason for exploring fused filament fabrication (FFF), which makes use of filament that is extruded layer by layer to form a three-dimensional object, was twofold [19]. The use of FFF over SLA printing is advantageous due to less work up, less waste, and less contamination. Furthermore, FFF provides the option for printing with copper, which is currently used as a heat sink and promotes heat flow through the solid–liquid interface. It was explored whether a cell which is fully manufactured of copper, and therefore has superior thermal conductivity, can enhance sensor sensitivity. However, FFF has some limitations inherent to its operation mechanism that involves extruding of molten polymers and stacking them layer by layer, which are discussed by Daminabo et al. [20]. Recently, this technique has progressed and it is possible to print filament with metal composites [21] and metal structures [22], which will improve the overall thermal conductivity of the system and thereby lead to a more uniform distribution of heat within the flow cell.

The heat dispersion in the 3D-printed measurement cells was studied with computational fluid dynamics with ANSYS software. The influence of key operating characteristics on the sensor performance was evaluated by measuring solutions of amocillin, a commonly used beta-lactam antibiotic [23], which has been linked to the accelerated development of antimicrobial resistance [24]. The new measurement design led to a twofold reduction in measurement time and a decrease in sample volume to 100 μL. Therefore, considering the affordability and straightforward mass production of additive manufacturing, this method has great potential for application in medical diagnostics.

## Experimental

### Reagents

Ammonium hydroxide, azoisobutyronitrile (AIBN), acrylamide, trimethylolpropane trimethacrylate (TRIM) and methanol were sourced from Sigma Aldrich (Gillingham, UK). 3-(Trimethoyxysilyl)propyl methacrylate and amoxicillin were provided by Acros Organics (Geel, Belgium). Phosphate buffered saline (PBS) tablets were purchased from Oxoid (Hampsire, UK). Hydrochloric acid, acetone, and toluene were acquired from Fisher (Loughborough, UK). Hydrogen peroxide (33% solution) was supplied by Alfa Aesar (Heysham, UK). All deionised water (DI) had a resistivity higher than 18 MΩ cm^−1^.

### Additive manufacturing of measurement cells

Flow cells were produced on a Form 2 stereolitography 3D-Printer (Formlabs, USA) using a Form 2 Clear Resin (GPCL04) and have an inner volume of ~ 100 μL. The design is shown in Supporting Information (S-1A) and is similar to ref [13], bar a slight increase in size (original design had 5 mm of height and 5.29 mm in diameter, the addition-type flow cell had 8 mm of height and 6 mm of diameter) and replacing the screws with bolts which poses less strain on the material. The increase in dimension led to less fluctuation in signal due to environmental changes and the use of bolts improved the overall shelf-life of the measurement set up. The requirements for the printing of the measurement cells were waterproof, high-quality prints within a reasonable time-frame using a method compatible with mass production.

**Fig. 1:**
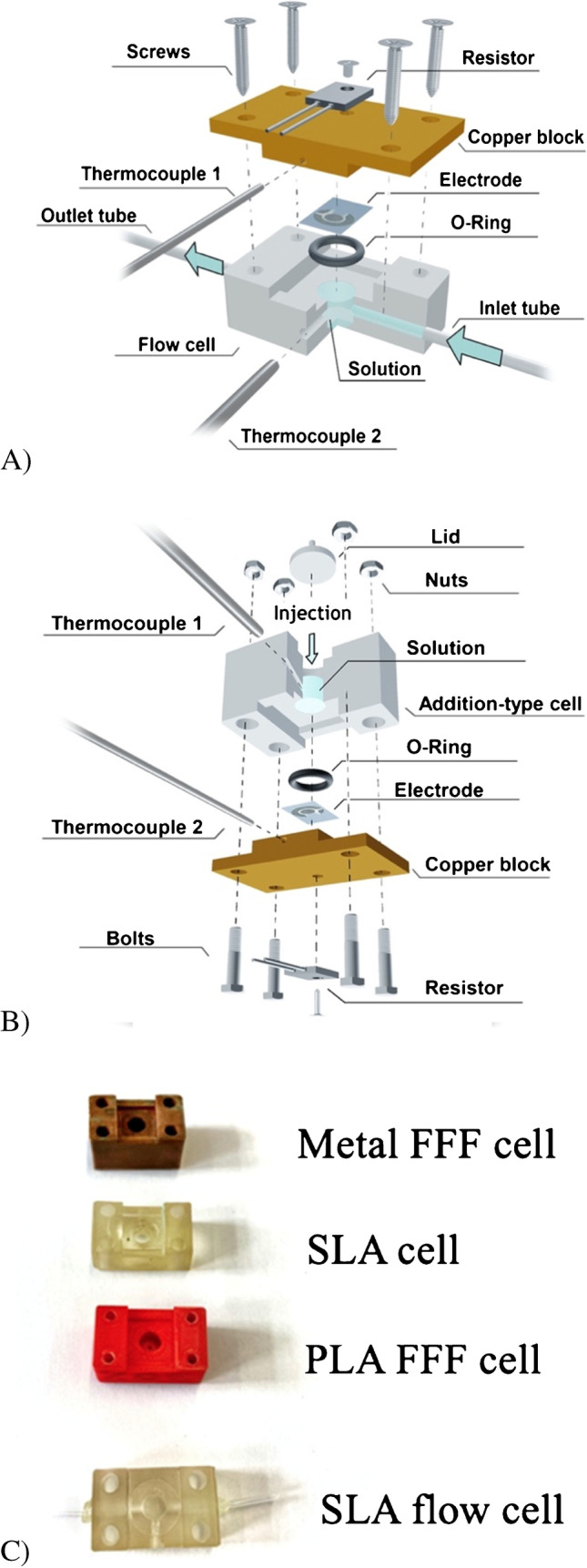
Schematic design of the original flow cell design (**A**) and a liquid reservoir where liquid sample is directly added via an inlet on the top (**B**). Blue arrows indicate direction of flow. Images were created with Blender version 2.92. The evolution of the different measurement designs and materials is shown in (**C**), with the bottom image demonstrating the different 3D printing measurement cells

Furthermore, to reduce the volume of the liquid reservoir, different designs were developed and produced with an Anycubic Photon S using their 405 rapid photopolymer resin for SLA printing. The differences between the two measurements shown up are schematically shown in Fig. 1 (A is flow cell, B is addition-type reservoir). For design B, a lid was 3D printed to seal off the cells and prevent evaporation. A full technical drawing with all correct dimensions can be found in Supporting Information S-1B. The different materials used for the designs are shown in Fig. 1C.

The inlet for the thermocouple provides a point of leaking for the liquid. To prevent this from occurring, the inlet for the thermocouple was positioned at an angle with respect to the horizontal, which also had the added benefit of enabling smaller sample volumes to be used by having the tip of the thermocouple (the sensitive part) pointing downwards.

Stereolitography was successful in producing the redesigned measurement cells (Fig. 1B). These cells were also manufactured by fused filament fabrication (FFF), a common method of 3D printing where the material is extruded from a heated nozzle layer by layer to create a 3D object [25]. It was attempted to print the FFF flow cells on the Raise3D Pro2 + machines using two materials: (Poly-lactic Acid) and PLActive (Nano-Copper enhanced PLA) [26]. However, due to consistent nozzle jams, potentially caused by the additional abrasiveness of the copper nano-composite filament, it was not possible to print the PLActive cells. Thus, only PLA FFF cells were produced. A consistent issue with the FFF prints was leakage from the cells; hence the printer settings were fine-tuned by trial and error. Various unsuccessful solutions were attempted to fix the leakage, including printing more outer shells and incorporating a 3D-printed thermoplastic polyurethane (TPU) bung to improve the seal at the entrances of the flow cells. It was found that printing cells with 100% infill (concentric pattern, Fig S2-A) with the outer layers in opposing directions (rectilinear pattern, Fig S2-B) created the flow cells with the least leakage. The cells were printed with an extrusion width of 105% relative to the nozzle to cause slight over-extrusion which helped fill the small gaps between the lines of material. A layer height of 0.15 mm was used as a compromise between print quality and time. Breakaway supports were also created and included in the total print time of 1 h 17 min per cell.

Finally, we produced the same measurement cells from copper and ceramic filaments to analyse how this impact the performance. The model files were uploaded to Eiger, Markforged’s cloud slicer, and oriented in such a way that the need for internal support was removed. In the slicing process, a 0.129-mm-layer height was set and 4 layers were defined with solid (100%) infill. The parts were allowed to scale for material shrinkage once sintered, meaning the main body of the cell was printed at 34.8 × 20.9 × 16.8 mm^3^ to create a 30 × 18 × 14.5 mm^3^ sintered copper piece. A build sheet was fixed to the vacuum build table and left to pre-heat. Copper and ceramic filaments from MarkForged Copper (Markforged, Massachussetts, US, https://copper3d.com/) were loaded into the heated chamber and given time for the brittle wax binder to soften before they could be loaded onto the hotend of the MetalX (metal X System, Markforged, Massachusetts, US). When removed from the printer, the parts were in the “green” state and were carefully placed into custom designed and printed TPU cartridges, designed to protect the delicate parts in transit. The prints were then washed for 15 h in Opteon SF79 (Opteon, Wilmington, US) to remove as much of the binder material as possible, leaving the part semi-porous to allow the remaining binder to burn off during the sintering. The parts were then dried and placed into a sintering kiln, forming a homogeneous copper part over approximately 25 h by bringing the prints close to the melting point of copper (930 °C) followed by slow cooling. This process turns the ceramic filament into a ceramic dust, allowing the parts to be removed easily using mechanical force or via sonication. Overall, the printing process for 4 cells was estimated at 1 day, with a wash time of ~ 16 h required.

### MIP and NIP syntheses

Glass slides (1 × 1 cm) were cleaned via ultra-sonication (5 min) in water, methanol and acetone sequentially. Once dried, the chips were immersed in a solution of hydrogen peroxide/hydrochloric acid/deionised (DI) water (1:1:5 respectively) and sonicated (for 20 min at 60 °C). The chips were then rinsed with DI water and the process was repeated similarly with the exception of the solution which was changed to hydrogen peroxide/ ammonium hydroxide/DI water ( 1:1:5 respectively). The chips, once thoroughly dried, were added to a 4% solution of 3-(Trimethoyxysilyl)propyl methacrylate in toluene. The chips were left on an orbital shaker (100 rpm) overnight to complete functionalisation. Finally, the chips were washed with toluene and dried.

MIPs were synthesized via dissolving amoxicillin (0.024 mmol), functional monomer acrylamide (0.11 mol), crosslinker monomer TRIM (0.20 mmol), and initiator AIBN (0.016 mmol) into 250 μL of DMSO. This composition is based on previous work demonstrating acrylamide is the most suitable monomer for amoxicillin detection [27]. After degassing with N_2_, 5 µL of the solution was placed onto a functionalized glass chip and polymerized by exposure to a UV lamp (Polytec UV LC-5 light source (λmax = 365 nm, Karlsbad, Germany) with a distance of 5 cm (light source to chip) for 1 min. The chips were washed for 10 s in chloroform and left to dry. The polymerisation and washing was repeated twice more to produce three polymer layers on the chip. The template was extracted from the polymerized chips with a 50% aqueous solution of methanol, which was refreshed several times during the 2 days the chips were agitated on an orbital shaker. UV-analysis was used to confirm that all traces of the template were removed from the polymer-functionalised chips. Non-imprinted polymers (NIPs) were synthesized according to the same protocol but without addition of the template to the monomer mixture.

The surface structure of the MIP-modified glass slide was evaluated by scanning electron microscopy (SEM). Figure S-4 shows a full homogeneous coverage of the glass-functionalised chip with porous polymer material. SEM measurements were recorded on a Supra 40VP (Carl Zeiss, Cambridge, UK) with an average chamber and gun vacuum of 1.3 × 10^–5^ and 1 × 10^–9^ mbar. To enhance the contrast, a thin layer of Au/Pd (8 V, 30 s) was sputtered onto the electrodes using a SCP7640 sputter coater (Polaron, Hertfordshire, UK).

### HTM measurements of MIP-functionalised glass slides

The MIP-modified glass squares (1 × 1 cm^2^) were pressed onto the copper block and mounted into the 3D-printed measurement cell of choice. These cells were then sealed with an O-ring and connected to a home-made HTM set up that was first described by van Grinsven et al*.* [4]. A resistor (22 Ω) was attached to the copper block, enabling it to act as a heat source that was actively steered with a Proportional-Integral-Derivative (PID) controller. The PID parameters had a significant impact on the noise of the signal and had to be optimised for each measurement cell as their heat transfer behaviour and consequently loss to the environment is different. Optimised PID settings are shown in Table 2 in the results.

The thermal resistance (R_th_) at the solid–liquid interface was monitored in order to evaluate binding of the target to the MIP layer. This was calculated by subtracting the temperature of the copper block (T_1_) over the temperature monitored in the liquid (T_2_) to determine the temperature gradient, which was subsequently divided by the power (P) to keep the heat sink at the set temperature of 37 ± 0.02 °C. The temperature in the liquid (T_2_) was measured every second with a thermocouple type K. Once binding of the target of interest occurs to the MIP layer, this results in a measurable decrease in T_2_ which leads to an overall increase of the thermal resistance (R_th_). The principle of this pore blocking model is schematically shown in Figure S-5. The 3D-printed sample holders containing MIP-functionalised electrodes were placed into an INCU-line VWR (Lutterworth, UK) incubator which allowed control of the ambient temperature surrounding the cells.

At first, measurements were conducted with an empty measurement cell (without MIP-functionalised electrodes) into which PBS was pipetted via the opening on the top. This was subsequently sealed off with a custom 3D-printed lid to prevent evaporation and the thermal resistance was monitored over time. Parameters that influenced the noise were assessed such as the temperature of the environment (by adapting the temperature of the incubator from 27, to 32–37 °C) and the parameters of the PID feedback loop. For subsequent measurements on amoxicillin, MIP-modified glass slides were mounted into the 3D-printed measurement set up and PBS was added. The system was left until a stable baseline was established and subsequently, the liquid in this flow cell was replaced with a PBS solution spiked with the target molecule amoxicillin (100 nM). This process was repeated for the different designs and compared in terms of stabilisation time, noise on the signal, and relative increase upon exposure to the target, to the original flow cell as described in [13]. For measurements on the flow cells, the liquid in the flow cell was added with a dual channel LSP04-1B programmable syringe pump from Longer Precision Pump Co., (Hebei, China) with a volume of 3 mL being injected at a flow rate of ~ 2 mm/min. To establish the specificity of the MIP sensors, identical measurements were performed with NIP-modified glass slides. For the best performing measurement set up, measurements with multiple PBS-spiked solutions with amoxicillin (0, 10, 100, 1000 nM) were conducted for both MIP and NIP.

### Computational fluid dynamic modelling

Computational fluid dynamic (CFD) modelling was undertaken using ANSYS 2021 R1 in order to study the fluid flow and heat transfer within the flow cells. This was performed for both the resin and copper flow cells, at three ambient temperatures (17, 27, 37 °C). The analysis was only performed for the flow-through type cell. The complexities of modelling an addition-type cell, particularly the air-fluid interactions (evaporation), are deemed beyond the scope of this study.

Transient simulations were performed using a time-step of 0.5 s, which was optimised in our previous study via a time-step independence study [13]. Standard mesh settings were used with an element size of 0.3 mm and automatic inflation with smooth transitions. This resulted in 127,941 mesh elements. This procedure was also optimised in our previous study [13] via a grid independence study, which used a similar mesh density.

The boundary conditions were defined as follows:

1. The heat source was the bottom surface and defined as a copper block with a constant temperature of 37 °C located 5 mm from the fluid domain (approximately mimicking the location of the control thermocouple in the experimental setup).
2. All other external walls were heat sinks whereby heat was lost via natural convection to air with an approximated film heat transfer coefficient of 25 Wm^−2^ K^−1^. The walls were defined as copper (thermal conductivity 401 Wm^−1^ K^−1^, Density 8940 kgm^−3^, Specific Heat capacity 385Jkg^−1^ K^−1^ as reported in the National Insitute of Standards and Technology (NIST) database) or resin (thermal conductivity 0.25 Wm^−1^ K^−1^, Density 1200 kgm^−3^, Specific Heat capacity 1110 Jkg^−1^ K^−1^ as available from vendor Clear Resin Technical datasheet), and the wall thickness was varied in different zones of the cell in order to mimic the geometry of the experimental cell. It should be noted that these were not conjugate simulations and that the material properties and thicknesses are used in the model solely for the purpose of calculating the thermal resistance of the heat sink boundaries.
3. All other surfaces were adiabatic.

Standard settings for modelling free convection in Fluent were used, as outlined in the user guide. This included the use of the Boussinesq approximation, which was suitable in this case due to the small temperature differences in the flow cell (ambient temperature of 17–37 °C and heat source of 37 °C). The fluid was modelled as pure water. Each simulation was run for a total of 600–1200 time steps, giving 300–600 s of simulation data per case and depending on the simulation time required to reach an approximate steady state as indicated by the levelling off of temperature and velocity fields. Each time step converged within 4–10 iterations. Solving each model took approximately 1 h, performed on a Windows PC utilising an AMD Ryzen 5 3600X 6-core processor with 12 threads clocked at 3.8 GHz (CPU) and 16 Gb of RAM at a speed of 3200 MHz. Velocity and temperature fields were exported from the simulation for post-processing in the ANSYS CFD-POST application.

## Results

### Thermal measurements in PBS

At first, it was necessary to determine the required liquid volume required in the addition-type cell in order to achieve stable measurement signals. To this end, water was pipetted into the resin reservoir with different volumes. When a volume of 90 μL was used, within 30 min, the resistance of air was measured rather than of the liquid, suggesting this is not sufficient to conduct temporal measurements. A stable baseline level was achieved when using sample volumes of 100 μL, which was further used for measurements with filament and resin-printed reservoirs (Figure S-6). A subsequent experiment focused on determining the optimal stabilisation time in addition to the impact on the noise on the signal when moving away from the original flow cell design. Instead of water, PBS was used since it mimics the ionic strength and pH (7.4) of blood.

The results are summarised in Table 1. Experiments were conducted at different environmental temperatures and PID settings. These experiments were conducted with the resin-printed and copper measurement cell, as initial results for the measurement cell produced by fused filament deposition had aforementioned issues with voiding, leading to sample leakage and high rate of evaporation. Even though the copper measurement cell had some issues in this respect since it relies on a similar production principle, this could easily be combatted by applying a coat of varnish on the exterior of the cells which was not possible with the filament measurement cells. Furthermore, the printer has an additional feature to adjust size and account for shrinkage, making voidage less likely.

**Table 1:** Baseline values and relative noise on signal

Experiment	Baseline R_th_ values (°C W^−1^)		
	Amb. T = 27 °C	Amb. T = 32 °C	Amb. T = 37 °C
SLA cell 1*	5.67 ± 0.05	5.74 ± 0.05	8.79 ± 0.12
SLA cell 2**	7.25 ± 0.05	6.50 ± 0.04	5.28 ± 0.18
FFU Metal Cell 1^***^	5.64 ± 0.04	5.54 ± 0.05	5.11 ± 0.10
FFF Metal Cell 2^****^	5.05 ± 0.03	5.71 ± 0.05	5.32 ± 0.06

*These cells were printer with the SLA Anycubic Photon printer and used PID settings: P1_I10_D0.3

**The same design as SLA cell 1, but with other PID settings: P1_I15_D0

***Copper cells printed with FFF, with PID settings: P1_I10_D0.3

****The same design as FFF Metal cell 1 but with different PID settings: P1_I15_D0

Figure S-7 provides an overview of reported relative noise signal in the literature of SLA-printed flow cells and flow cells manufactured (drilled) from Perspex. While these experiments were conducted with different substrates, it is expected that the origin of the noise tends to be electronic and therefore does not have a significant impact on the average standard deviation. In general, literature reports that the average standard deviation on the signal tends to be between 0.7 and 1%. From Table 1, the average deviation on the signal can be calculated which for the resin is ~ 0.9% at 32 °C (P = 1, I = 10, D = 0.3) and could be further optimised to ~ 0.6% by adjusting the PID settings to P = 1, I = 15, D = 0. For the copper flow cell, there is no considerable difference between the PID settings and it remains around 1%. Therefore, it was demonstrated that both the measurement cells exhibited a stable signal and are suitable for thermal measurements and have potential to improve the signal to noise ratio compared to the original flow cell design.

The influence of the ambient temperature was also studied. In general, there was a trend towards lower R_th_ values with increasing environmental temperatures. This can be explained by the fact that a smaller temperature difference with the environment leads to less heat loss and therefore a lower overall temperature gradient (T_1_ – T_2_), resulting in a reduction of the R_th_. Since we report the signal as a relative increase to the baseline, it would be beneficial to have lower R_th_ values. However, when the incubator was kept at the same temperature as the measurement cell, this led to overcompensation by the PID feedback loop, which therefore made it not suitable for measurements. Pre-heating of the samples therefore did not improve response time; thus, a balance needs to be struck between the incubator temperature and keeping a sufficient temperature gradient for the PID feedback to rapidly correct the measured temperature to the set value.

The PID parameters did not have a significant impact on noise. However, it is known they do have a considerable impact on start-up times and therefore we further investigated how the material characteristics and feedback loop affect the time until stabilisation. This is an important consideration for the commercial prospect of the sensor platform considering measurement times should ideally not exceed 5 min for point-of-care applications.

Table 2 summarises the time to reach steady state for the different measurement cells, which was defined as the time when the average reading of the thermocouple varied by less than 0.1ºC degree over a 1-min period. It was not possible to reach a stable signal for SLA cell 2 at 37 °C since the PID loop needs a temperature gradient in order to stabilise the signal.

**Table 2:** Time until steady state for the different measurement cells

Experiment	Ambient = 27 °C	Ambient = 32 °C	Ambient = 37 °C
SLA cell 1 ^*^	800 s	900 s	900 s
SLA cell 2^**^	1200 s	450 s	-
FFF metal cell 1^***^	1200 s	900 s	800 s
FFF metal cell 2^****^	850 s	900 s	1300 s

*These cells were printer with the SLA Anycubic Photon printer and used PID settings: P1_I10_D0.3

**The same design as SLA cell 1, but with other PID settings: P1_I15_D0

***Copper cells printed with FFF, with PID settings: P1_I10_D0.3

****The same design as FFF Metal cell 1 but with different PID settings: P1_I15_D0

In a previous research, stabilisation times of at least 30 min (1800s) were considered prior to injecting samples, which limited the point-of-care application of the sensor. Modelling studies confirmed this was due to the velocity of the liquid, which leads to disturbance in the signal and therefore prolongs the stabilisation period [13]. When samples are simply pipetted in, the velocity of the liquid is virtually zero and in all scenarios, the stabilisation time does not exceed 20 min. While it was anticipated that the copper flow cells would stabilise faster due to having a higher thermal mass, this was not the case. A likely explanation for this is that directing the heat flow via one copper sink is faster compared to heating being distributed all over the cell.

While the impact of the PID parameters was not very clear with regard to reducing the overall noise, the I (integral) component played a vital role in the response time. For instance, we determined that by changing the I from 10 to 15 led to improving the time until steady state from 900 to 450 s. Therefore, it is key to adapt the PID settings depending on the material and design of the measurement cell used prior to each measurement in order to optimal sensor performance.

### Computational fluid modelling

**Fig. 2:**
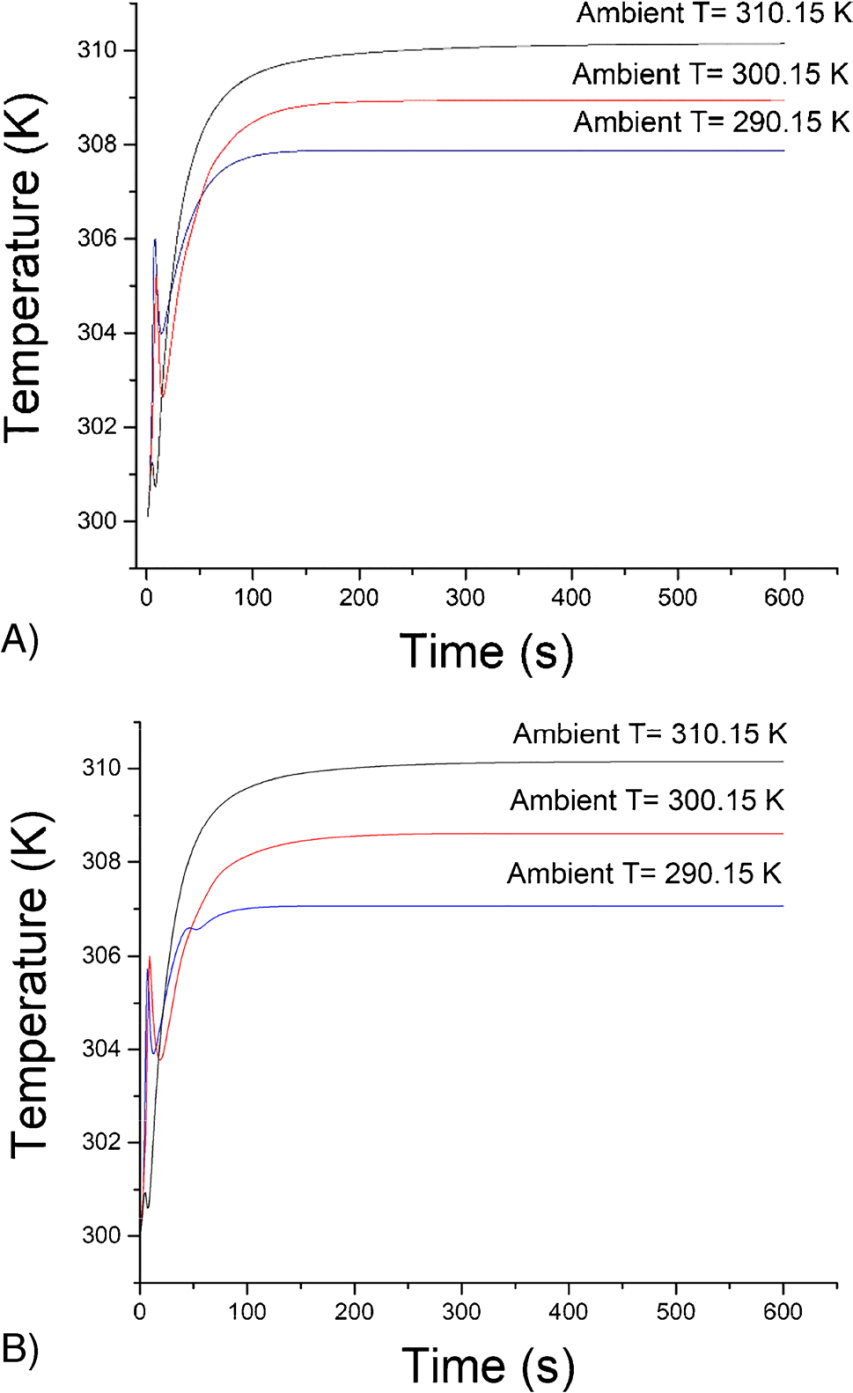
Temperature of the fluid at the thermocouple tip vs time at three ambient temperatures, with 37 °C being the dop dashed line, 27 °C the middle dotted line, and 17 °C the lower solid line for (**A**) SLA cell and (**B**) copper cell

Figure 2 shows the temperature of the fluid in contact with the thermocouple tip vs time, and as such gives an indication of the expected settling time of the sensor measurement. It was demonstrated that there is negligible difference between the copper and resin cells, which was largely in agreement with the experimental results. A lower ambient temperature led to a lower temperature of the liquid, which was in line with the performed measurements. Lower ambient temperatures were expected to lead to slightly faster settling times, but experimentally, no clear trend was found.

**Fig. 3:**
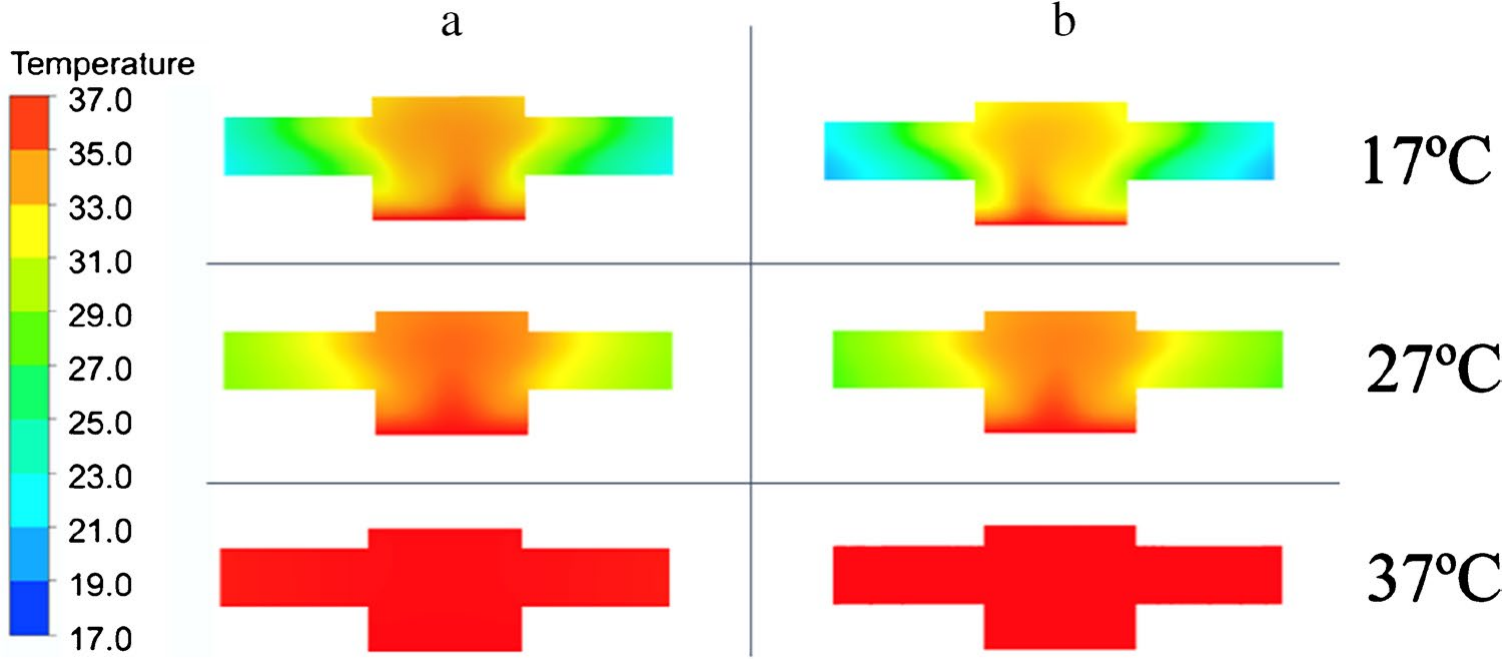
Temperature contour plots at steady state at different ambient temperatures. a: SLA printed flow cell, b: Metal (copper) measurement cell

An indication of signal noise for each flow cell can be obtained by studying temperature contour plots and the average velocity in the cell: if the fluid temperature in the cell significantly varies and a velocity is present, the signal will be affected by fluid of varying temperature flowing across the thermocouple. Figure 3 shows the steady-state temperature contours for both the resin and copper cells at various ambient temperatures on a consistent temperature scale. The ambient temperature had a significant influence on the temperature within the cell while the material of construction had no significant influence. For both materials, the fluid temperature within the flow cell (central section) varies by around 4 °C for an ambient of 17 °C, 1–2 °C for an ambient of 27 °C and with no variation at all for the 37 °C ambient case (due to the environment and heater block being at a constant temperature). Hence, it was expected that measurement cells situated within an ambient environment similar to that of the heater block (in this case 37 °C) would encounter less noise than those in colder environments. This was further supported by the cell velocity magnitude data shown in Figure S-8. As natural convection flows are driven by the temperature difference between source and sink (heat block and ambient temperature), the velocity magnitude reduces with an increase in the ambient temperature, to 0 mm/s for the case of a 37 °C ambient. A lower velocity in the cell should correlate to decreased noise, as there is less refresh of the fluid in contact with the thermocouple. In general, lower noise was found in measurements at 32ºC compared to 27ºC, which was in agreement with the modelling. However, when the incubator was set at the same temperature as the programmed temperature of the heat sink, the feedback loop is not able to control the signal leading to high noise on the signal. Thus, it was found that 32ºC was the optimal ambient temperature for measurements.

The computational fluid modelling results show that lower ambient temperatures resulted in faster settling times, while higher ambient temperatures would lead to less noise on the signal. However, the experimental results do not follow such clear patterns suggesting that other aspects of the flow cell design (e.g. the temperature controller) have a greater impact on the signal stability compared to the underlying fluid dynamics and heat flow in the cell.

### Thermal measurements of MIP-functionalised electrodes.

**Fig. 4:**
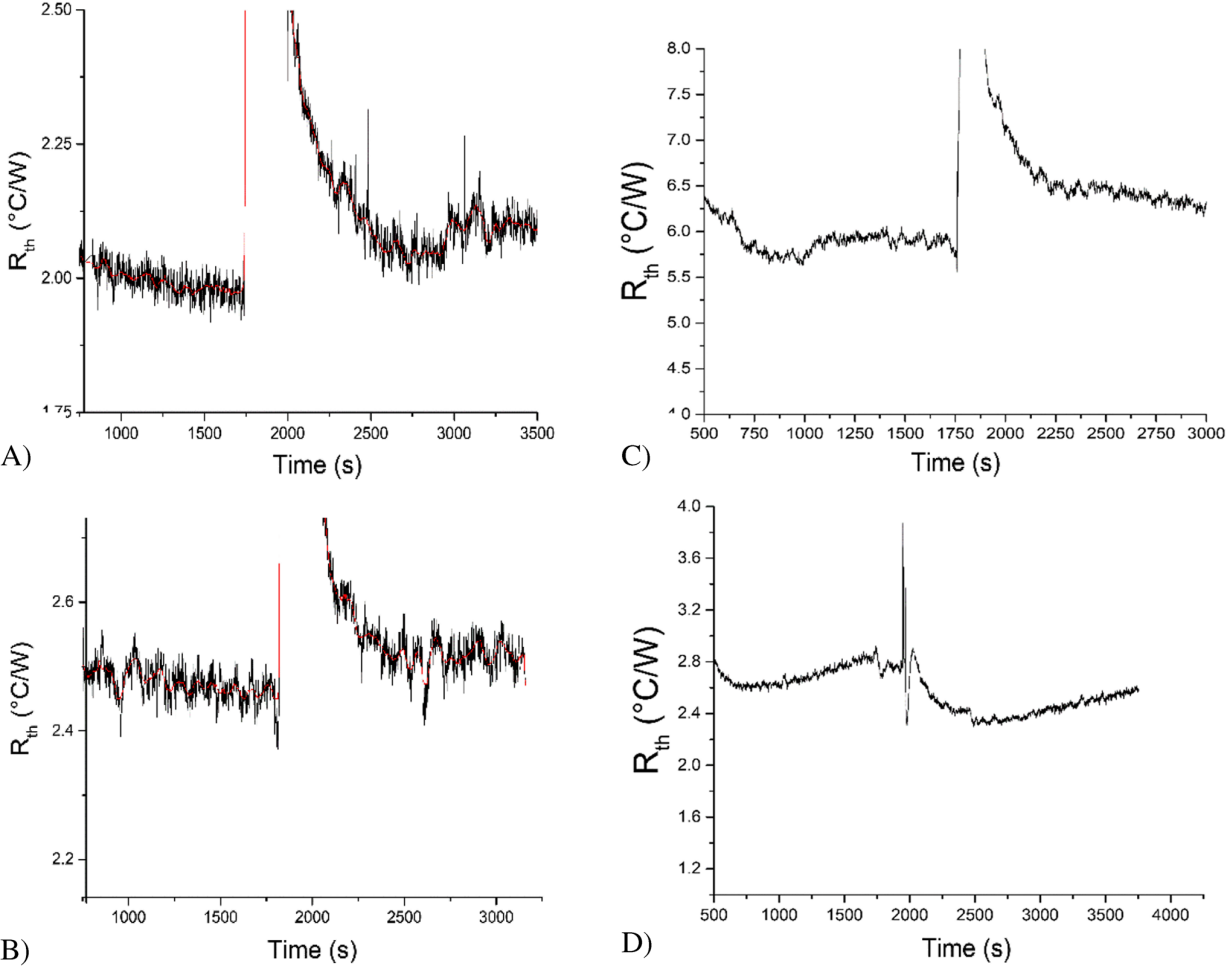
Thermal measurements performed with SLA (**A**) measurement cell, FFF (**B**) measurement cell, SLA flow cell design (**C**) and metal FFF (**D**) measurement cell. In all cells, a MIP-modified electrode for amoxicillin was inserted. These cells were stabilised in PBS for 1750s (cell A, B, D) and 2000s (cell C) after which a PBS solution spiked with 100 nM of amoxicillin was added. The red line represents a gentle median filter (average taken over 50 points) on the data

MIP layers were functionalised onto the glass slides as described in 2.3. Scanning electron microscopy analysis (SEM) confirmed there was full and uniform coverage of the surface, as shown in Figure S-4. These MIP-modified glass slides were inserted into the measurement cells and stabilised in PBS for approximately 30 min after which a solution of 100-nM amoxicillin in PBS was injected. This process was repeated for all measurement cells including the original flow-through design, as shown in Fig. 4. To assess the specificity of the sensor, measurements were performed with a reference NIP, which results are shown in Figure S-9 and indicate that no increase in thermal resistance was found when NIPs were exposed to solutions spiked with amoxicillin. The copper measurement had a lower thermal resistance compared to the other cells, which can be explained by the higher conductivity of the material. This will ultimately lead to a smaller temperature gradient and minimal changes in signal when samples are added. There was also difficulty in repeating measurements with this design as the lower level of heat loss to the environment led to a higher rate of evaporation, which was observed in some cases as a continuous upwards drift of the signal.

Figure 4 clearly demonstrates the difference in stabilisation time between the addition-type set up and the flow-cell design. All addition cells are stabilised between 450 and 1000 s, whereas for the flow-cell design at least 1500 s was needed. In fact, in general at least two injections with PBS are performed (Fig. 3c shows signal after second PBS injection) to ensure the signal was stable before the measurement was amoxicillin was conducted. The addition cells also required a considerably shorter time (750 s) to stabilise after the samples are added via pipetting, thus having a much faster measurement time compared to the flow cell that needed at least 1500 s or more to record a reliable value. Increasing the speed of injection will not help in that respect as a certain residence time is needed to achieve binding between target and polymer.

While one example was shown for the FFF PLA cell, it was very difficult to repeat this measurement, and the reported increase was not found to be significant. We also encountered issues with the FFF metal cell; whilst it was possible to combat leakage by applying varnish to the outside of the cell, a coat of varnish might also need to be applied to the inside. Another option is to add slightly more liquid to the measurement chamber (200 vs 100 μL**)** since some of the liquid will be absorbed by the pores inside the flow chamber. Despite repeating these measurements several times, no significant response was found with copper as material for the flow cell. A possible explanation for this could be that a temperature gradient is necessary to analyse heat flow at the interface, whereas the FFF metals have an even heat distribution across the cell walls. Table 3 summarises the results for the four different designs, comparing the differences in measurements. The measurement with the cells printed with SLA was performed in triplicate and had error bars of around 1% for the relative response to amoxicillin and ± 100 s for the stabilisation time.

**Table 3:** Summary of the different parameters for the designs, conducted with optimised PID settings (see Table 2)

Material	3D printer	Noise on signal (%)	Relative response at c = 100 nM (%)	Sample volume (μL)	Stabilisation time (s)
SLA cell	Anycubic Photon	~ 0.6	6.5 ± 1	3000	450
FFF PLA cell	Raise3D Pro2 Plus	~ 2	2.5	100	1000
SLA flow cell	Anycubic Photon	~ 1	4.5	100	1800
FFF metal cell	Markforged	~ 1	n/a	200	900

The SLA resin addition cell and SLA flow cell are user-friendly, circumvent issues with voiding, and are both suitable for thermal analysis of antibiotics with MIP-based sensors. The addition-type measurement cell demonstrated a similar response at 100 nM compared to previous research where MIP-modified screen-printed electrodes were used [28]. These designs can be applied in different areas; for instance, an addition-type design due to its low sample volume and shorter stabilisation time will have its merit in measuring clinical samples. However, the flow cell design will be advantageous for high throughput of samples or if one is interested in studying dynamic systems.

**Fig. 5:**
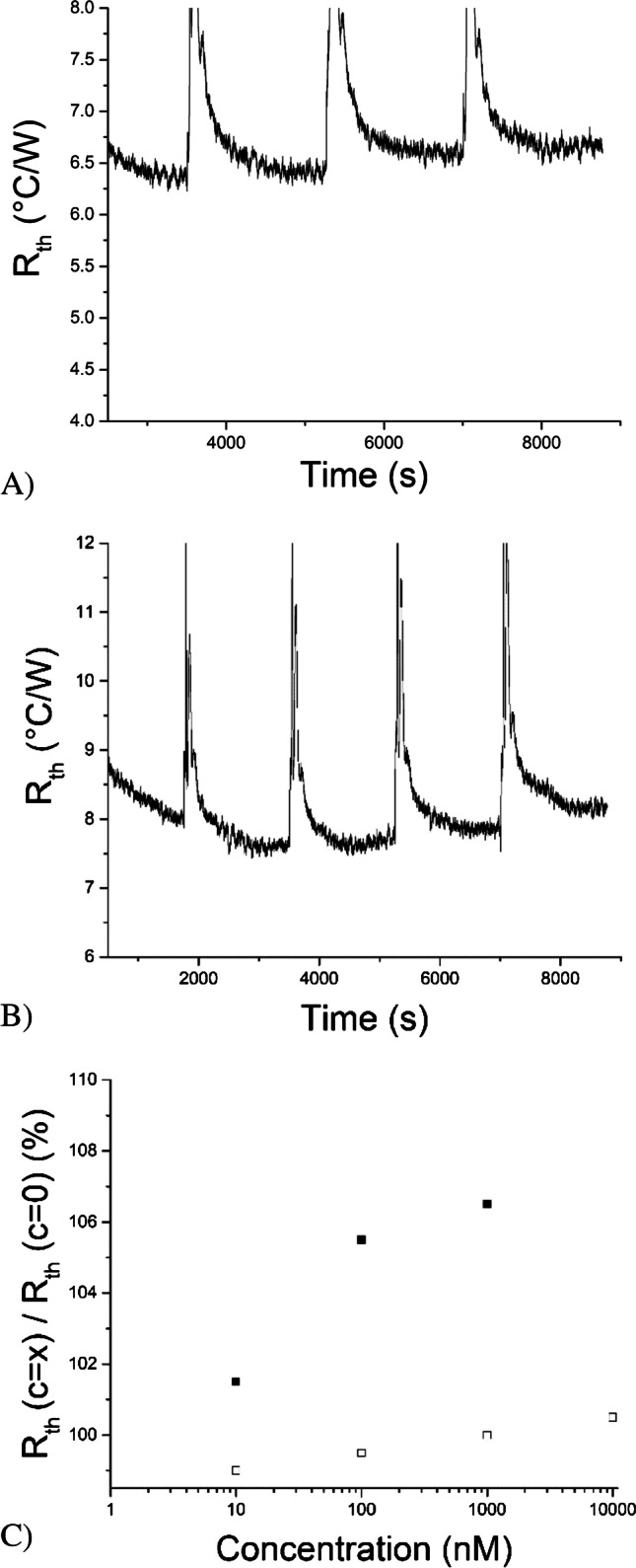
(**A**) Thermal resistance over time when MIP-modified electrodes mounted into a SLA measurement cell were exposed to PBS solutions spiked with amoxicillin. (**B**) Thermal resistance over time wh;en NIP-modified electrodes mounted into a SLA measurement cell were exposed to PBS solutions spiked with amoxicillin. (**C**) Normalised change in R_th_ for MIP (filled squares) and NIP (open squares) upon exposure to PBS spiked solutions with amoxicillin (0, 10, 100, 1000 nM for MIP, 0–10, 100, 1000, 10,000 nM for NIP), demonstrating the MIP are able to specifically capture amoxicillin.

To establish proof-of-application of the system, further measurements were conducted with the SLA cell which demonstrated the highest potential for future applications (see Fig. 5). The results for both MIP (Fig. 5a) and NIP (Fig. 5b) are shown, with a comparison of the relative R_th_ response upon exposure to PBS solutions spiked with amoxicillin (Fig. 5c). The measurements were repeated in triplicate with freshly prepared MIP-functionalised electrodes with a standard deviation of ~ 1% at respective concentrations.

There were some minor differences in absolute R_th_ values of MIP and NIP, which is likely linked to changes in structure (MIP structure is more porous) and film thickness. Once the thermal response was normalised, it was clear that only for the MIP a significant increase in R_th_ was observed which can be attributed to binding to the polymer layer. Using the three sigma method, the limit of detection was estimated to be ~ 10 nM, which is well below the tolerated maximum residue level of amoxicillin. In the future, dose–response curves will need to be established for determining amoxicillin content in water and environmental samples.

## Conclusions

A new CAD design of a measurement cell for thermal analysis was developed, from a flow cell design to an open addition set-up, in order to reduce sample volume nearly 20-fold whilst also reducing stabilisation time. These measurement cells were produced using three different materials, respectively, PLA filament, photoresin, and copper, and three different additively manufacturing methods including FFF, SLA, and FFF in combination with MIM. The performance of the measurement cells was evaluated with measurements in buffered solutions and by evaluating the binding of amoxicillin, an antibiotic, to a MIP-based functionalised sensor. There were significant differences in other key performance indicators, including cost, printing time, and ease of operation which is significantly affected by the void content. These findings suggest the 3D printing approach and not the material properties are crucial in designing measurement cells for thermal analysis (see Table 4).

**Table 4:** Key performance indicators for different 3D printing approaches. For FFF PLA was used as filament, FFF in combination with MIM was printed with copper powder, and SLA was printed with photoresin

3D printing approach	Average stabilisation time	Print time	Print costs	Material strength	Material void content
FFF	≈ 15–20 min	< 1 h	£0.3	Weak	High
SLA	5–10 min	2.5 h	£0.7	Sturdy	None
FFF & MIM	≈ 15 min	6.5 h	£14.0	Hard metal	High

Whilst FFF printing with PLA as the material has a fast printing time and low cost, it is not suitable for thermal analysis due to the high void content. Even when FFF was combined with MIM with a ceramic copper filament, voidage remained albeit this was somewhat reduced by taking shrinkage into account in the design and coating with varnish. However, the measurement cell made of copper is less suitable for point-of-care measurements due to the slow distribution of heat within the cell, which was confirmed by computational fluid dynamic modelling. Furthermore, printing time and cost were significantly higher compared to measurement cells produced by SLA. Therefore, the SLA design has the highest commercial potential for future applications and was further explored with MIP-based electrode measurements. Proof-of-concept of the system was provided by analysing a series of buffered solutions spiked with trace levels of amoxicillin, a prevalent antibiotic. Due to the high robustness of the MIPs and ease of operation of the proposed thermal set up, in the future this might provide the opportunity to measure contaminated water samples on-site which is not possible with existing sensors. This will facilitate rapid and low-cost detection of potential sources of antibiotic contamination, which is an important measure to reduce accelerated spread of antimicrobial resistance. However, more work is required to benchmark the sensor against existing assays, and analysis in real samples needs to be conducted to demonstrate proof of application.

## Supplementary Information

Below is the link to the electronic supplementary material.

Supplementary file1 (DOCX 2607 KB)
