# Supplementary material for: Influence of design and material characteristics on 3D printed flow-cells for heat transfer-based analytical devices
Source: Mikrochim Acta. 2022 Jan 24;189(2):73. doi: 10.1007/s00604-022-05163-2 (PMC8786792; doi:10.1007/s00604-022-05163-2)
Supplement: Supplementary file 1 — Supplementary file1 (DOCX 2607 KB) [file 604_2022_5163_MOESM1_ESM.docx]

**Electronic Supplementary Material**

**Influence of design and material characteristics on 3D printed flow-cells for heat-transfer based analytical devices**

Leonardo F Figueiredo ^1+^, Felipe S Vieira^2+^, Oliver D Jamieson^3^, Jack Reeder^3^, Thomas Mc Lean^3^, Jennifer Olsen ^3^, Robert D Crapnell ^4^, Matthew J Whittingham ^4^, Craig E Banks ^4^, Richard Law ^3^, Jonas Gruber ^5^, and Marloes Peeters^3*^

*1) Faculdade de Ciências Farmacêuticas, Universidade de São Paulo, Av. Prof. Lineu Prestes, 580, CEP 05508-000 São Paulo, SP, Brazil.*

*2) Departamento de Engenharia Química, Escola Politécnica, Universidade de São Paulo, Avenida Prof. Luciano Gualberto, trav. 3, 380, CEP 05508-900 São Paulo, SP, Brazil.*

*3) Newcastle University, School of Engineering, Merz Court, Claremont Road, NE1 7RU, Newcastle Upon Tyne*

*4) Manchester Metropolitan University, Faculty of Science and Engineering, John Dalton Building, Chester Street, M1 5GD, Manchester, United Kingdom.*

*5) Departamento de Química Fundamental, Instituto de Química, Universidade de São Paulo, Av. Prof. Lineu Prestes, 748, CEP 05508-000 São Paulo, SP, Brazil.*

**First author +:** L.F. Figueiredo and F.S. Vieira have contributed equally to this research paper and therefore will equally share first authorship.

**Corresponding author*:** Dr. Marloes Peeters (marloes.peeters@newcastle.ac.uk)

Newcastle University, School of Engineering, Merz Court, Claremont Road, NE1 7RU, Newcastle upon Tyne, UK

**Supporting Information S-1, Technical drawings of measurement cells**

**
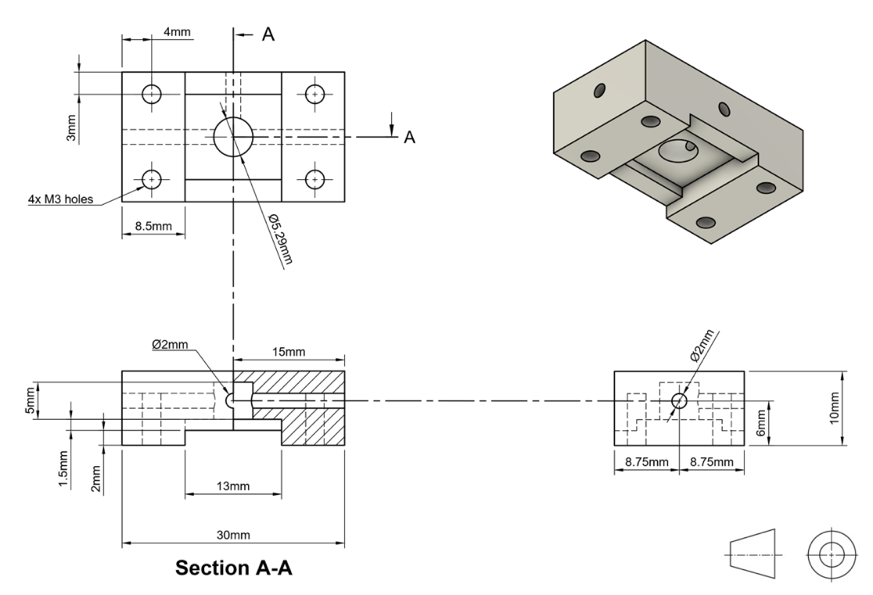
**

**A)**

**
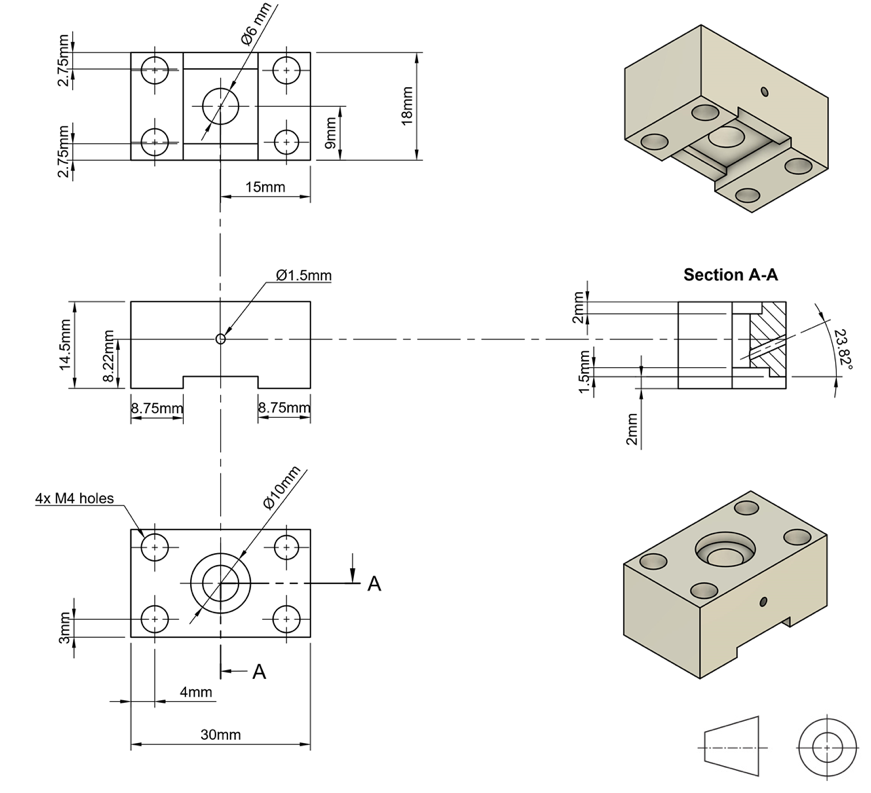
**

**B)**

**Figure S-1.** Technical drawings, which include dimensions, of the design for the original flow cell design (A) and off the novel addition-type measurement cell (B).

**Supporting Information, S-2:**


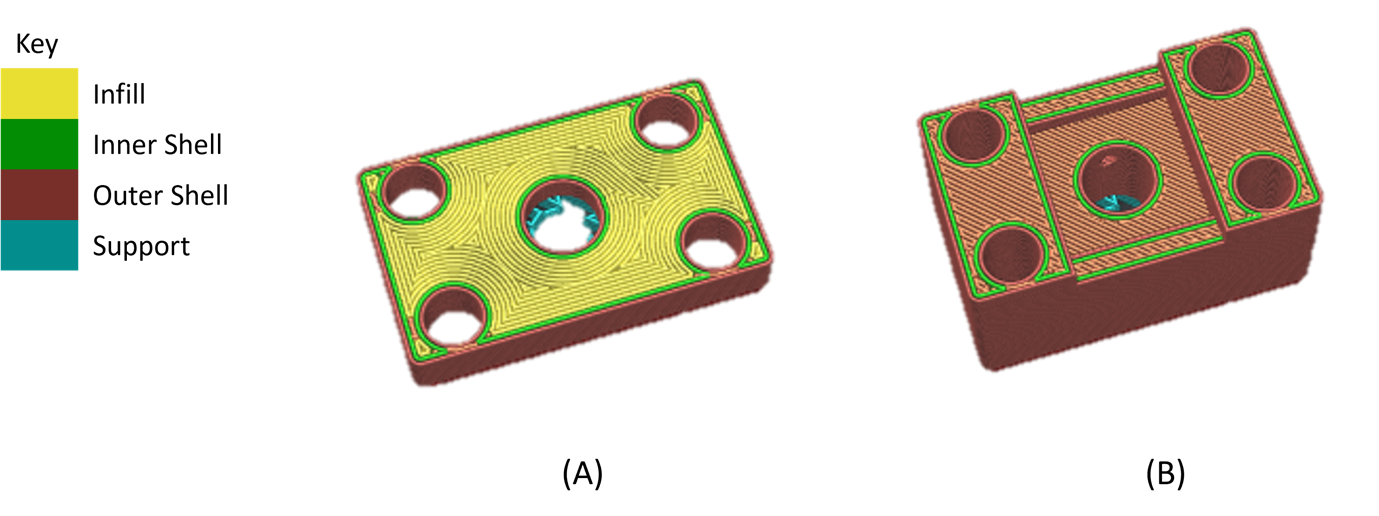


**Figure S-2.**Computer-aided design (CAD) design of the FFF PLA-printed cell. (A) A slice of the flowcell CAD file showing the concentric infill pattern. (B) The outer shell of the flowcell CAD file showing the rectilinear print pattern to enhance waterproofing.

**Supporting Information, S-3: FFF Metal printed cells.**


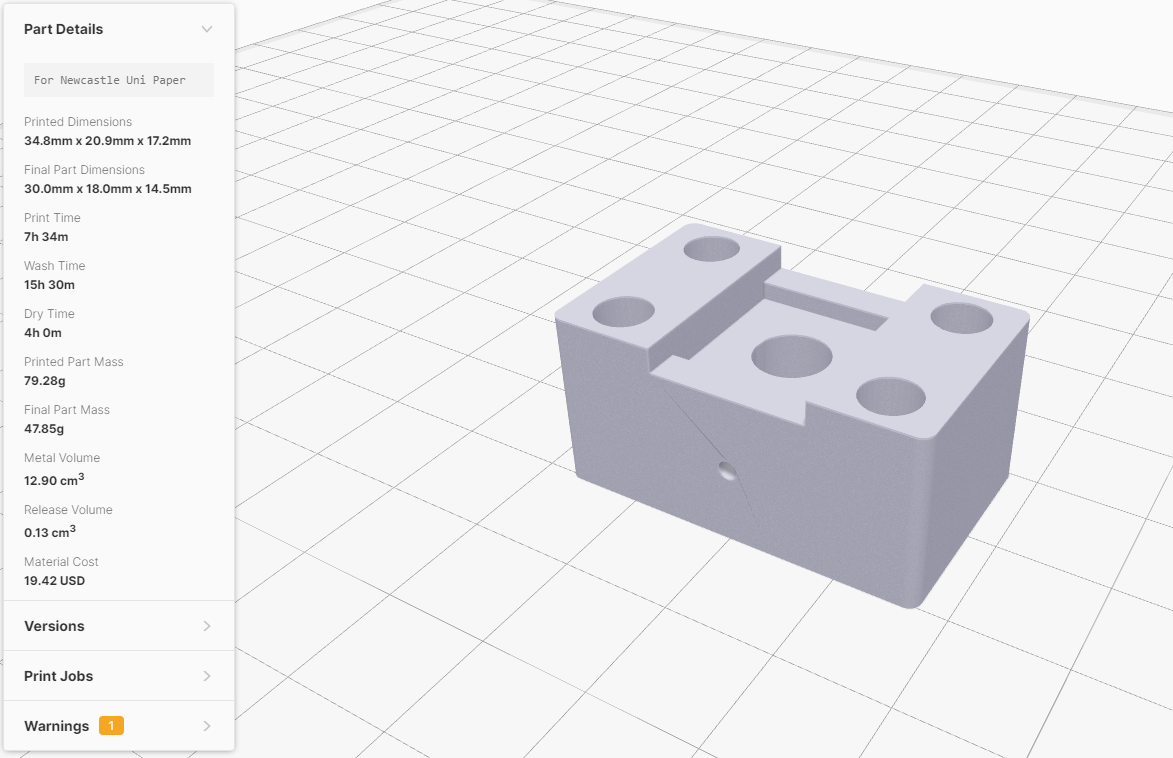


**Figure S-3.** Slicer (Markforged Eiger) Image of 3D-Printed Copper Cell with Print, Wash & Dry times – Including mass of final piece vs printed piece.

**Figure S-4: Scanning electron microscopy analysis of the MIP-modified glass slides**

**
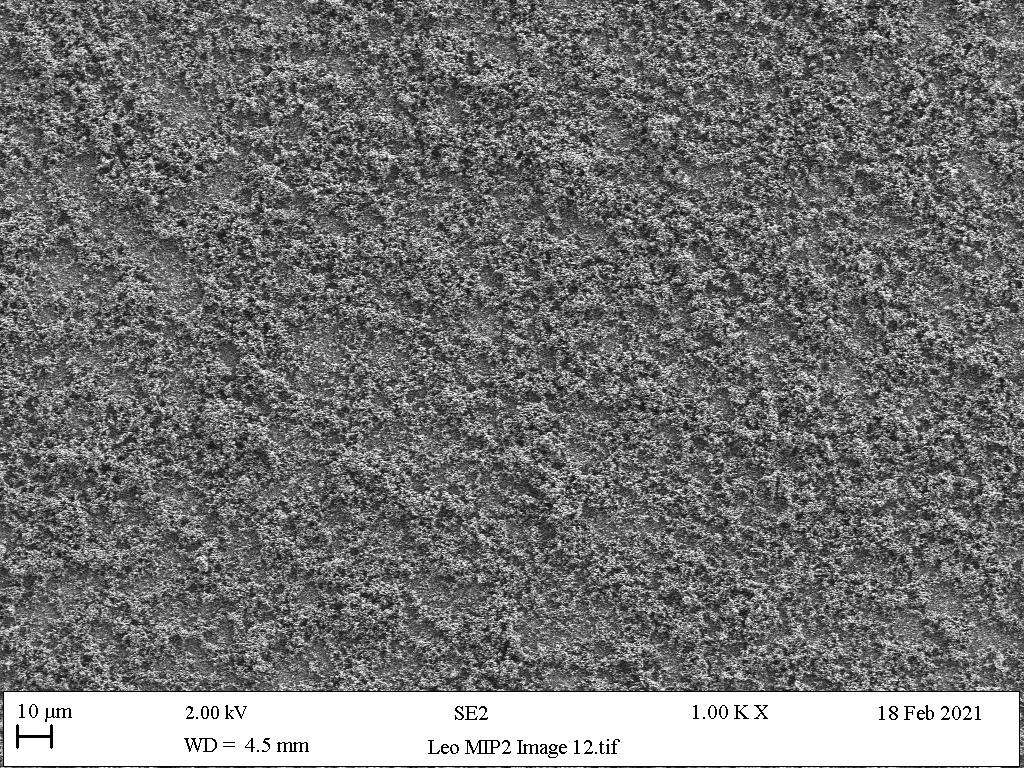
**

**Figure S-4**: A typical SEM image of the glass slides functionalised with MIP layers, indicating full and equal coverage of the surface with polymer material.

**Figure S-5: Schematic representation of the pore blocking model**


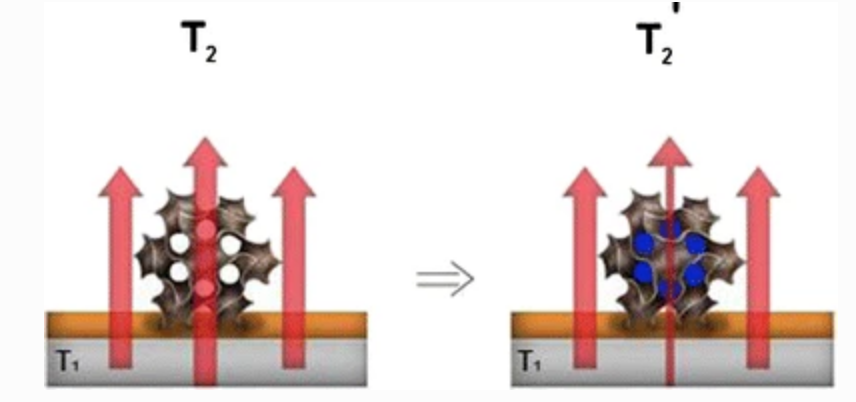


**Figure S-5**: The HTM device steers temperature T_1_ of the copper via a PID controller, whereas the temperature in the liquid (T_2_) is solely monitored (both measured with thermocouples type K). Binding of the target to the MIP layer reduces the heat flow through the surface, leading to an increase in the overall resistance of the solid-liquid interface and a measurable decrease in T_2_.

**Figure S-6: Experiments to study volume of measurement cell**


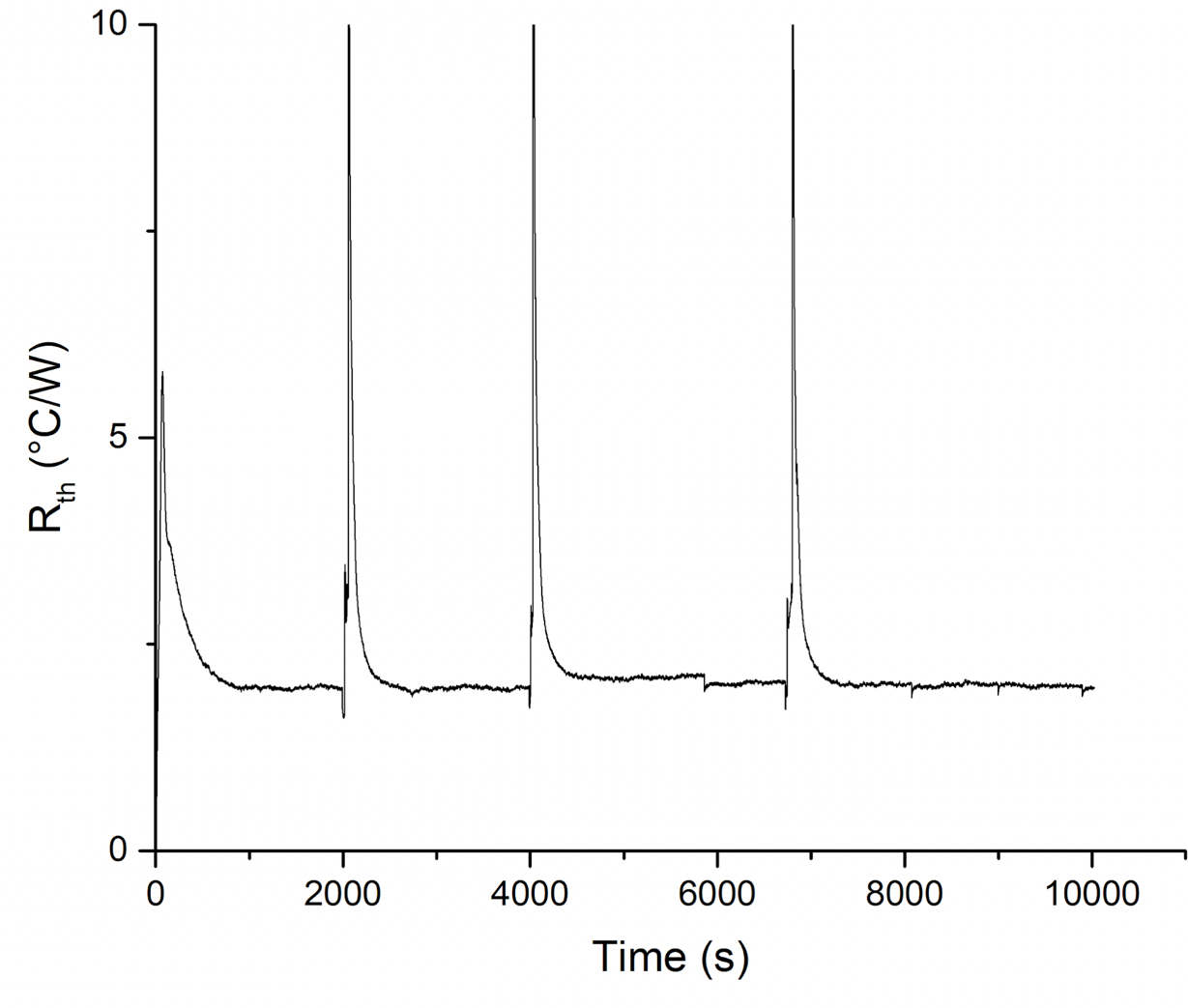


**Figure S-6:** Different volumes of water, respectively 130, 120, 110 and 100 µL were pipetted into the flow cell to estimate what volume is required. No significant differences in signal were seen here, indicating it is possible to conduct measurements with volumes as low as 100 µL. If lower volumes were employed there notably higher thermal resistance were measured over time, which indicates the thermocouple is not positioned into the liquid but measures air instead.

**Figure S-7: Literature references of different measurement designs**

| **Measurement cell** | **R_th_ ± SD Baseline (°C W^-1^)** | **Relative noise (%)** | **Reference** |
| --- | --- | --- | --- |
| **SLA flow cell**  Screen-Printed Electrodes (SPEs) | 4.29 ± 0.04 | 0.93 | [7] |
| **SLA flow cell**  Multiplex thermocouple design | 3.38 ± 0.03 | 0.89 | [13] |
| **Perspex flow cell**  SPEs printed onto polyvinylchloride and polyester substrate | 3.83 ± 0.02  4.12 ± 0.03 | 0.52  0.73 | [34] |

**Figure S-7:** Overview of reports in literature using thermal analysis for detection of organic compounds using either SLA printing to produce flow cells or drilling from Perspex. The average value of the thermal resistance was reported, which depends on the substrate material used, and average noise as this has an influence on the S/N ratio. It can be seen that the average noise of the signal was in the same range as what was measured with the new design.

**Supporting Information S-8: Cell velocity magnitude data**





**Figure S-8:** Cell velocity magnitude data as predicted via Ansys for different environmental temperatures, ranging from 17°C (top line) to 27°C (middle dashed line) to 37°C (lower solid line). The higher the temperature difference is between the liquid in the measurement cell and the environmental temperature, the higher the cell velocity is which has an impact on noise on the signal.

**Supporting Information S-9 – NIP measurement results:**

1.


2.



C)






D)

Figure S-9. Measurements were conducted with corresponding NIPs for amoxicillin. First, the cells were stabilised into PBS followed by an injection of amoxicillin at ~2000 s followed by a washing step with PBS at ~4000 s for scenario B and C. The panel demonstrates the SLA cell (A), FFF printed PLA cell (B), SLA printed flow cell (C), and metal FFF cell (D). It was clear that the FFF printed PLA cell demonstrates significantly higher noise compared to the SLA printed cells (2% noise vs <1%). The FFF metal printed cell showed little noise but there was a somewhat upwards drift, which can be explained by potential evaporation of the liquid.
